# Real-world treatment patterns and clinical outcomes among patients with diffuse large B-cell lymphoma in a US healthcare claims database

**DOI:** 10.1038/s41408-025-01412-8

**Published:** 2025-12-05

**Authors:** Mazyar Shadman, Jennifer S. Harper, Alex Bokun, Chang Xu, Pei Lin, Gloria Graf, Xiaoxiao Lu

**Affiliations:** 1 Clinical Research Division, Fred Hutchinson Cancer Center, Seattle, WA USA; 2 Division of Hematology and Oncology, University of Washington, Seattle, WA USA; 3 Johnson & Johnson, Titusville, NJ USA; 4 Johnson & Johnson, Horsham, PA USA; 5 Johnson & Johnson, Raritan, NJ USA

**Keywords:** Cancer, Cancer

## Abstract

Treatment options for diffuse large B-cell lymphoma (DLBCL) have expanded, but real-world data on treatment patterns and outcomes remain limited. This study examined real-world outcomes in DLBCL patients treated between 10/1/2015 and 6/30/2024. Patients were stratified by lines of therapy (LOT) and treatments (1L rituximab, cyclophosphamide, doxorubicin, vincristine, prednisone [R-CHOP]; 2L stem cell transplant [SCT]; and chimeric antigen receptor T-cell [CAR T] therapy (any LOT). Variables were reported descriptively. Time-to-event outcomes were assessed using the Kaplan-Meier method. LOT data from 9875 patients were included. R-CHOP-based regimens were the most common 1L treatment (61.7%–67.3% in 2016–2023; 49.4% in 2024). Conventional chemoimmunotherapy use decreased in 2L (81.6% in 2016 to 41.9% in 2024) and 3L (47.6% in 2016 to 22.1% in 2024), while novel therapies increased (43.0% in 2L and 55.9% in 3L in 2024). Median overall survival declined across LOT (1L: 58.1 months; 2L: 30.0 months), as did median time to next treatment (1L: 36.1 months; 2L: 10.6 months). Twelve-month treatment failure rates were 36.0% after 1L, 51.8% after 2L, and 42.2% after CAR T. Among CAR T recipients, 93 received one of 36 distinct subsequent regimens, indicating no standard of care. These findings highlight the unmet needs in DLBCL.

## Introduction

Non-Hodgkin lymphoma (NHL) is the most common hematological malignancy, with an estimated 80,620 new cases in the United States (US) in 2024 [1]. Diffuse large B-cell lymphoma (DLBCL) is the most common type of NHL, accounting for approximately 25%–30% of all NHL cases [2]. Since the early 2000s, the R-CHOP combination (rituximab, R; cyclophosphamide, C; doxorubicin, H; vincristine, O; and prednisone, P) has been the standard first-line (1L) treatment for newly diagnosed DLBCL [3–5].

While approximately 60% of patients treated with R-CHOP achieve long-term remission, a notable proportion of patients experience relapse or become refractory to the initial treatment [6]. For years, these patients had limited treatment options and a poor prognosis.

However, the treatment landscape for DLBCL has evolved with the introduction of novel therapies, including chimeric antigen receptor T-cell (CAR T) therapies, bispecific antibodies, and other targeted therapies [7–9]. In 2023, the US Food and Drug Administration (FDA) approved polatuzumab vedotin (pola) in combination with rituximab, cyclophosphamide, doxorubicin, and prednisone (R-CHP) as a 1L treatment for DLBCL based on findings from the POLARIX study [10]. These advancements have drastically expanded treatment options for both newly diagnosed and relapsed or refractory DLBCL.

Despite these therapeutic advancements, real-world research suggests high treatment failure rates and poor survival outcomes, particularly in patients requiring later lines of therapy (LOT) [11–13]. Furthermore, recent large-scale real-world data on treatment patterns and clinical outcomes across different LOT in DLBCL, especially before and after CAR T, remain limited. To address this knowledge gap, we conducted an updated evaluation of real-world treatment patterns, particularly the adoption of novel therapies, and clinical outcomes in adult patients with DLBCL in the US, using a large claims database.

## Methods

### Study design and data source

**Fig. 1:**
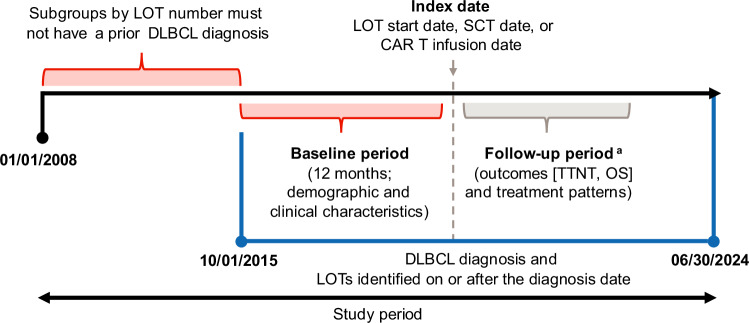
Study design. CAR T chimeric antigen receptor T-cell, DLBCL diffuse large B-cell lymphoma, LOT line of therapy, SCT stem cell transplant, TTNT time to next treatment or death, OS overall survival. ^a^Follow-up period was defined as the time from the index date to death, end of continuous enrollment, or end of study period,whichever occurred the earliest.

This retrospective observational cohort analysis used Optum’s de-identified Clinformatics^®^ Data Mart database (Fig. 1). The database contains de-identified data from more than 180 million US individuals with private health insurance (commercial and Medicare Advantage plans) and represents a geographically diverse patient population spanning all 50 states. Core data include demographics, claims for inpatient and outpatient services, prescriptions, and mortality (validated using stringent patient-matching criteria). This comprehensive dataset provides a robust sample size, enhancing the generalizability of the findings to patients across the US.

The database used for this study is HIPAA-compliant. This study used de-identified claims data and was exempt from Institutional Review Board (IRB) review.

### Line of therapy identification

LOT for DLBCL were identified using an algorithm similar to previously published DLBCL LOT algorithms [14, 15]. The first step in this algorithm was to identify the initial DLBCL diagnosis (International Classification of Diseases, 10^th^ Revision, Clinical Modification [ICD-10-CM] code C83.3x; effective October 1, 2015), followed by LOT identification. 1L started with the first DLBCL therapy administered following DLBCL diagnosis, with combination regimens including all DLBCL therapies initiated within 30 days of the start of 1L. LOT advanced when one of the following occurred: a new DLBCL therapy was observed >30 days after the start of a LOT, stem cell transplant (SCT) was observed, or a regimen was discontinued and restarted after >90 days. Maintenance therapies, salvage therapies (considered as the same LOT as transplant), and bridging therapies (considered part of the same LOT as CAR T) did not advance LOT. Radiotherapy was excluded from the LOT algorithm and was not considered in this study.

### Study population

This LOT-level study included adult patients with DLBCL in the Optum Clinformatics^®^ database who had one or more LOT. The index date per LOT was the LOT start date (except for LOT including stem cell transplant [SCT] or CAR T, where the index date was the SCT or CAR T infusion date). LOT were further required to meet all of the following criteria: a diagnosis with DLBCL (International Classification of Diseases, 10th Revision, Clinical Modification [ICD-10-CM] code C83.3x) on at least (1) one inpatient encounter with DLBCL as the primary diagnosis or (2) two outpatient encounters at least 30 days apart before or on the index date; ≥365 days of continuous health care enrollment before and ≥29 days after the index date; and an age of ≥18 on the index date. This study excluded LOT with a clinical trial code during the 6-month period before or on the index date, or a diagnosis of certain cancers (i.e., other NHL or non-NHL hematologic malignancies) within 12 months before the index date. The follow-up period was from the index date to the earliest of the end of continuous health plan enrollment, death, or the end of the study period.

Patients’ LOTs meeting the study criteria were stratified and assessed by LOT number (1L, 2L, and 3L) and specific treatments, including 1L R-CHOP (R-CHO observed; corticosteroids were ignored as they may be under-captured in claims data), 2L SCT, CAR T (any LOT), and post-CAR T (any LOT). For the 1L, 2L, 3L, 1L R-CHOP, and 2L SCT subgroups, the following two additional requirements were applied for more accurate LOT classification: (1) continuous enrollment starting ≥6 months before the first observed DLBCL diagnosis (washout period) and through the index date; and (2) patients with ICD-9-CM codes starting with 200.7 at any time before the DLBCL diagnosis date were excluded to account for potentially prevalent cases of DLBCL with potential treatment prior to the ICD-10 era. Because the CAR T and post-CAR T subgroups were not stratified by LOT, these two additional requirements were not applied to those subgroups.

### Data analysis

Patient demographics, clinical characteristics, and treatment patterns were described descriptively. Continuous variables were reported using mean, standard deviation (SD), and median, while categorical variables were presented as frequencies and percentages.

1L, 2L, and 3L treatment regimens were classified into 12 groups using the following hierarchy: (1) CAR T; (2) SCT; (3) pola + R-CHP (contains at least pola, R, C, and H; may include other drugs); (4) pola ± other; (5) R-CHOP (contains at least R, C, H, and O; may include other drugs); (6) tafasitamab (tafa) ± other; (7) R monotherapy; (8) chemotherapy; (9) R-squared (contains at least R and lenalidomide); (10) other (contains at least lenalidomide, ibrutinib, venetoclax, or selinexor); (11) immunotherapy (does not contain chemotherapies); and (12) chemoimmunotherapy (all other therapies). Corticosteroids were not considered during classification.

Overall survival (OS), time to next treatment or death (TTNT) as a proxy for duration of response, and treatment failure rates (defined as the percentage of patients who initiated a next LOT or died within 12 or 24 months) were evaluated using Kaplan–Meier survival analyses. Per Optum’s deidentification policies, patient counts <5 were masked.

## Results

### Patient demographics and characteristics

**Fig. 2:**
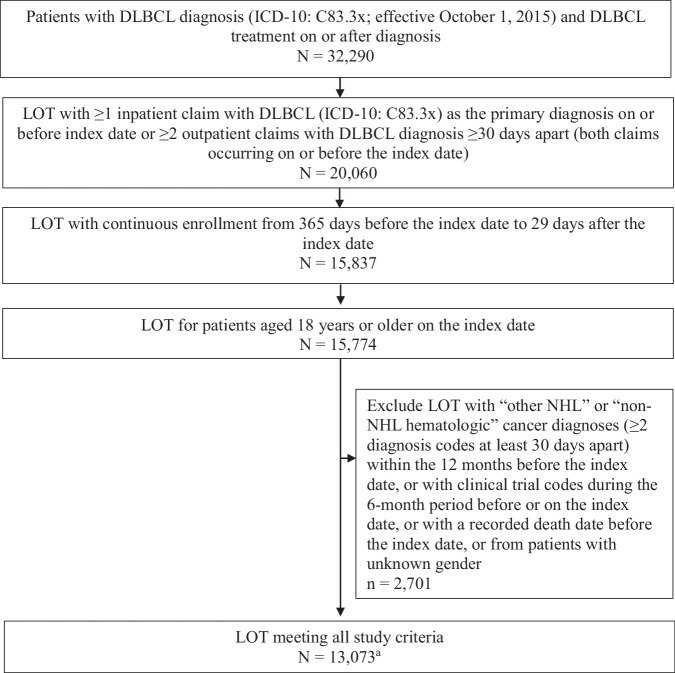
LOT identification. DLBCL diffuse large B-cell lymphoma, ICD International Classification of Diseases, LOT line of therapy, NHL non-Hodgkin lymphoma. ^a^Corresponding number of patients: 9875.

A total of 9875 patients had LOT that met the study criteria (Fig. 2). Patient demographics and baseline clinical characteristics for the 1L, 2L, and 3L subgroups are summarized in Table 1. Characteristics of patients treated with CAR T are provided in Supplementary Table 1. The demographic composition remained largely consistent across the LOT. In 1L, the mean patient age was 71.6 years; 53.8% of patients were male, 70.1% were White, 78.8% were covered by Medicare Advantage, and 21.2% had commercial health insurance. The median Quan-Charlson comorbidity index (QCCI) was 4.0 in 1L and 5.0 in 2L, and 3L. Across 1L, 2L, and 3L, infections (79.2% in 1L), hypertension (76.7% in 1L), and anemia (58.7% in 1L) were the 3 most common comorbidities.

**Table 1:** Demographic and clinical characteristics of patients with DLBCL.

	1L n = 7522	2L n = 2602	3L n = 880
**Demographics**			
Age at index, years			
Mean (SD)	71.6 (11.8)	71.4 (11.4)	71.4 (11.5)
Age category, n (%)			
<65 years	1502 (20.0)	584 (22.4)	209 (23.8)
65–69 years	1041 (13.8)	347 (13.3)	105 (11.9)
70–74 years	1593 (21.2)	546 (21.0)	169 (19.2)
≥75 years	3386 (45.0)	1125 (43.2)	397 (45.1)
Sex, n (%)			
Male	4050 (53.8)	1456 (56.0)	488 (55.5)
Female	3472 (46.2)	1146 (44.0)	392 (44.5)
Race, n (%)			
White	5272 (70.1)	1865 (71.7)	623 (70.8)
Black	514 (6.8)	148 (5.7)	52 (5.9)
Asian	229 (3.0)	74 (2.8)	28 (3.2)
Other or unknown	1,507 (20.0)	515 (19.8)	177 (20.1)
US region, n (%)			
South	2,980 (39.6)	1,019 (39.2)	348 (39.5)
Northeast	1,073 (14.3)	357 (13.7)	133 (15.1)
Midwest	1880 (25.0)	701 (26.9)	222 (25.2)
West	1568 (20.8)	522 (20.1)	174 (19.8)
Unknown	21 (<1)	^a^	^a^
Payer type, n (%)			
Commercial	1592 (21.2)	624 (24.0)	225 (25.6)
Medicare advantage	5927 (78.8)	1978 (76.0)	655 (74.4)
Unknown	^a^	^a^	^a^
**Clinical characteristics**			
QCCI			
Mean (SD)	5.0 (2.8)	5.3 (2.8)	5.3 (2.8)
Median	4.0	5.0	5.0
Comorbidities, n (%)			
Infection	5954 (79.2)	2098 (80.6)	704 (80.0)
Hypertension	5773 (76.7)	1934 (74.3)	651 (74.0)
Anemia	4418 (58.7)	1765 (67.8)	647 (73.5)
Fatigue or asthenia	3320 (44.1)	1368 (52.6)	490 (55.7)
Anxiety or depression	2613 (34.7)	973 (37.4)	342 (38.9)
Coronary artery disease	2402 (31.9)	876 (33.7)	294 (33.4)
Obesity	2286 (30.4)	746 (28.7)	218 (24.8)
Neutropenia	1383 (18.4)	1074 (41.3)	423 (48.1)
Thrombocytopenia	1308 (17.4)	728 (28.0)	324 (36.8)
Time from first observed diagnosis to index date, months			
Mean (SD)	3.4 (8.4)	13.7 (15.2)	22.6 (17.6)
Median	1.3	8.0	17.0
Follow-up time, months			
Mean (SD)	24.0 (22.9)	19.2 (20.0)	15.5 (16.7)
Median	15.9	11.5	9.1

Patients may be included in multiple subgroups. The 1L subgroup is smaller than the overall 9875 patients due to additional requirements for subgroup analysis by LOT number.

*1L* first line, *2L* second line, *3L* third line, *DLBCL* diffuse large B-cell lymphoma, *QCCI* Quan-Charlson Comorbidity Index, *SD* standard deviation, *US* United States.

^a^Per Optum’s deidentification policies, patient counts <5 were masked.

The median time from DLBCL diagnosis to the 1L index date was 1.3 months. The median follow-up duration shortened with each LOT, decreasing from 15.9 months after the 1L index date to 11.5 after the 2L index date and 9.1 months after the 3 L index date.

### Distribution of 1L, 2L, and 3L treatment regimens

**Fig. 3:**
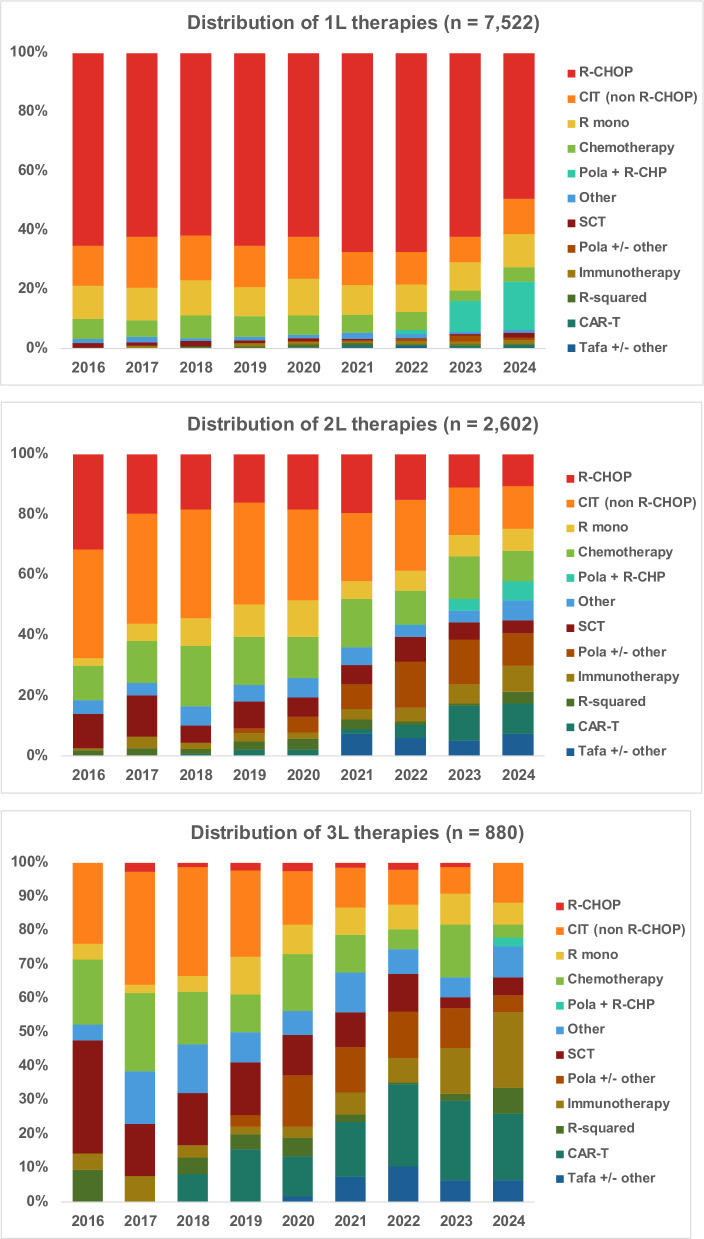
Distribution of therapies among patients with DLBCL by year. Time period: January 1, 2016 to June 30, 2024. A single patient may contribute to multiple lines of therapy. 1L first line, 2L second line, 3L third line, CAR T chimeric antigen receptor T-cell, CIT chemoimmunotherapy, DLBCL diffuse large B-cell lymphoma, IT immunotherapy, mono, monotherapy, Pola polatuzumab vedotin, R-CHOP rituximab cyclophosphamide doxorubicin and vincristine (with or without corticosteroids), R-CHP rituximab cyclophosphamide and doxorubicin (with or without corticosteroids), R-squared rituximab and lenalidomide, SCT stem cell transplant, Tafa tafasitamab.

1L, 2L, and 3L treatment regimens during the study period were grouped into the 12 categories shown in Fig. 3. R-CHOP remained the most common 1L therapy throughout the study period, with 65.3% of patients receiving an R-CHOP-based regimen and another 13.4% receiving non-R-CHOP chemoimmunotherapy (CIT) regimens in 2016 (Supplementary Table 2). The distribution of 1L therapies remained consistent until 2023–2024, when the use of pola plus R-CHP-based regimens began to increase. In 2L, the use of conventional CIT and chemotherapy without immunotherapy decreased from 81.6% in 2016 to 41.9% by the end of June 2024; the use of SCT also decreased, from 11.4% to 4.3%. During the same period, the use of pola-, tafa-, CAR T-, and other novel immunotherapy-based regimens (such as bispecific antibodies and antibody-drug conjugates) steadily increased, with 43.0% of 2L patients receiving one of these therapies in the first half of 2024. In 3L, the use of conventional CIT and chemotherapy without immunotherapy declined from 47.6% in 2016 to 22.1% from January to June 2024. By the first half of 2024, 55.9% of 3L patients were treated with CAR T-, pola-, tafa-based, and other novel immunotherapy regimens.

### CAR T subgroup

A total of 420 patients received CAR T, including axicabtagene ciloleucel (axi-cel; 35.9%), lisocabtagene maraleucel (liso-cel; 24.5%), tisagenlecleucel (tisa-cel; 6.4%), and unspecified CAR T brands (33.3%; based on claim codes applicable to any CAR T brand). Demographic and clinical characteristics of patients with DLBCL treated with CAR T are provided in Supplementary Table 1. Among the 238 CAR T patients (56.7%) with an apheresis claim, the median time from apheresis to CAR T infusion was 33 days. Bridging therapies were observed in 157 patients (66.0%). The most common (defined as ≥5%) bridging regimens excluding corticosteroids included pola plus rituximab (13.4%), pola plus bendamustine and rituximab (6.7%), and rituximab monotherapy (5.9%; Table 2).

**Table 2:** Treatments before and after CAR T.

Number of patients receiving CAR T in any LOT	420	
**Before CAR T**^a^		
Patients with an apheresis claim, n (%)		238 (100)
Time from apheresis to CAR T infusion, days		
Mean		36.0
Median		33.0
Most common drug classes within bridging period, n (%)^b,c^		
Monoclonal antibody		95 (39.9)
Chemotherapy		75 (31.5)
Antibody-drug conjugate		62 (26.1)
Most common individual drugs within bridging period (before identifying combination regimens), n (%)^b,c^		
Rituximab		94 (39.5)
Polatuzumab vedotin		61 (25.6)
Cyclophosphamide		22 (9.2)
Gemcitabine		21 (8.8)
Oxaliplatin		20 (8.4)
Bendamustine		19 (8.0)
Methotrexate		13 (5.5)
Most common bridging regimens, n (%)^b,c^		
Pola + rituximab		32 (13.4)
Pola + BR		16 (6.7)
Rituximab monotherapy		14 (5.9)
**After CAR T**^d^		
Most common regimens, n (%)^b,c^		
Loncastuximab tesirine		11 (12.0)
Epcoritamab		11 (12.0)
Rituximab monotherapy		8 (8.7)
Pola + BR		7 (7.6)
Pola + rituximab		6 (6.5)
CAR T		5 (5.4)
Most common drug classes, n (%)^b,c^		
Monoclonal antibody		50 (53.8)
Antibody-drug conjugate		29 (31.2)
Chemotherapy		21 (22.6)
Other^e^		17 (18.3)
Bispecific antibody		14 (15.1)
CAR T		5 (5.4)

*BR* bendamustine + rituximab, *CAR T* chimeric antigen receptor T-cell, *LOT* line of therapy, *Pola* polatuzumab vedotin.

^a^Bridging period: time from apheresis to 7 days before CAR T infusion, among the 238 patients with a claim for apheresis.

^b^Most common is defined as a frequency occurring in ≥5% of patients.

^c^Excludes corticosteroids.

^d^The next LOT following CAR T, among 93 patients with continuous enrollment from the CAR T index date through at least 29 days after the start of the subsequent LOT.

^e^Includes lenalidomide, ibrutinib, selinexor, and venetoclax.

After CAR T, 93 patients went on to receive one of 36 distinct regimens as the next LOT, indicating the absence of a standard of care post-CAR T. The leading drug classes post-CAR T, excluding corticosteroids, included monoclonal antibodies (53.8%), antibody-drug conjugates (31.2%), and chemotherapy agents (22.6%, Table 2). The most common regimens (defined as ≥5%) after CAR T included loncastuximab tesirine (12.0%), epcoritamab (12.0%), rituximab monotherapy (8.7%), pola plus bendamustine and rituximab (7.6%), pola plus rituximab (6.5%), and CAR T (5.4%).

### Clinical outcomes

#### OS and TTNT by LOT

**Fig. 4:**
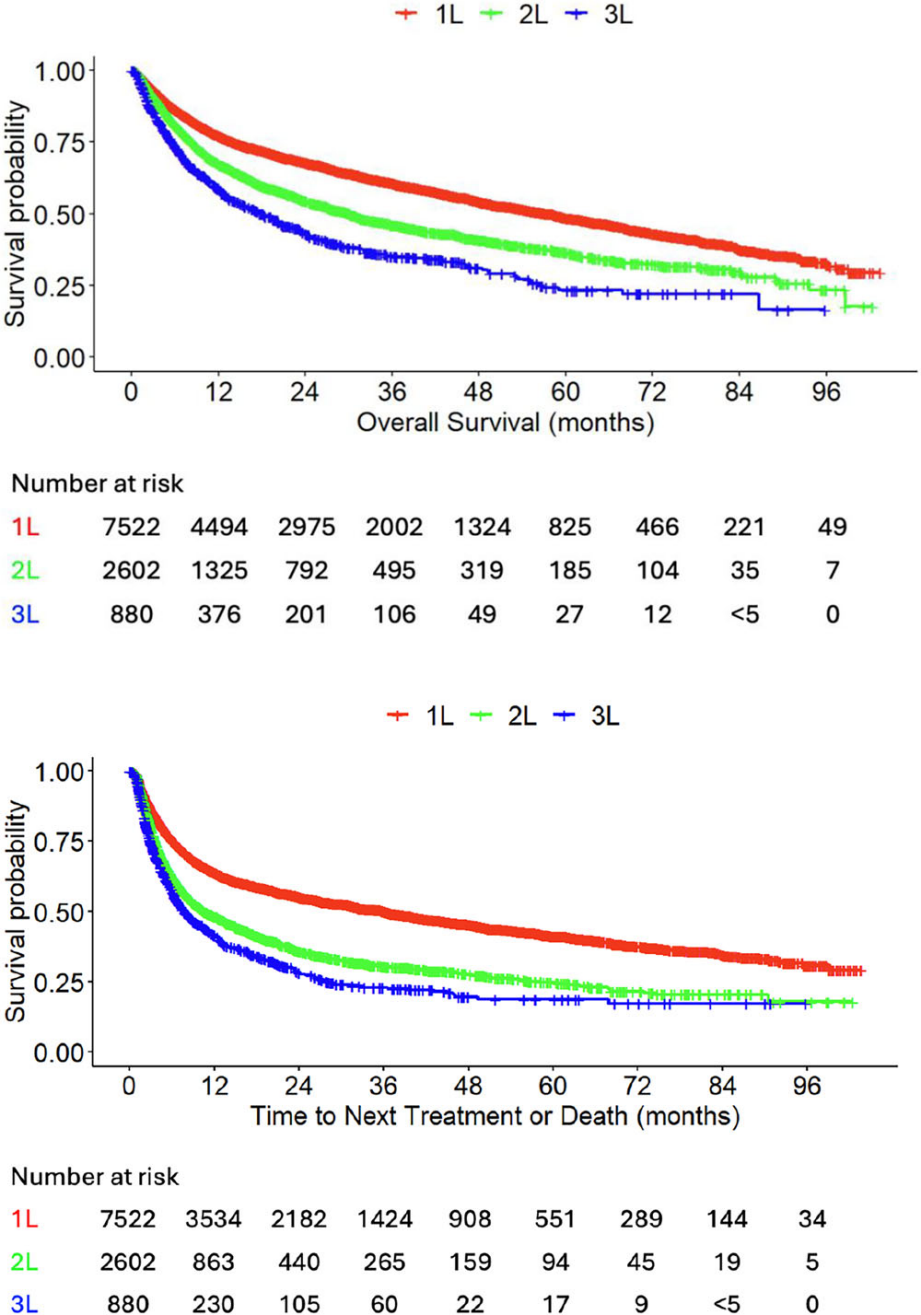
OS and TTNT by LOT. Per Optum’s deidentification policies, patient counts <5 were masked. 1L first line, 2L second line, 3L third line, LOT line of therapy, OS overall survival, TTNT time to next treatment or death.

During the study period, the median OS decreased across LOT, with 58.1 months (95% CI, 54.2–60.9) observed in 1L, 30.0 months (95% CI, 27.0–33.0) in 2L, and 18.2 months (95% CI, 15.2–20.9) in 3L (Table 3 and Fig. 4). Similarly, median TTNT declined substantially across LOT, from 36.1 months (95% CI, 32.5–38.9) in 1L to 10.6 months (95% CI, 9.6–12.5) in 2L, and 7.8 months (95% CI, 6.9–9.3) in 3L (Table 3 and Fig. 4).

**Table 3:** Clinical outcomes by LOT and treatment.

	Patient count	TTNT, months	OS, months	Failure rate within 12 months, %	Failure rate within 24 months, %
	n	Median (95% CI)	Median (95% CI)	Median (95% CI)	Median (95% CI)
LOT					
1L	7522	36.1 (32.5–38.9)	58.1 (54.2–60.9)	36.0 (34.9–37.2)	44.8 (43.5–46.1)
2L	2602	10.6 (9.6–12.5)	30.0 (27.0–33.0)	51.8 (49.6–53.8)	64.2 (61.9–66.3)
3L	880	7.8 (6.9–9.3)	18.2 (15.2–20.9)	58.3 (54.5–61.7)	72.0 (68.1–75.5)
Treatment					
1L R-CHOP	4277	58.0 (50.9–65.6)	68.2 (63.3–72.7)	29.2 (27.8–30.7)	37.5 (35.8–39.1)
2L SCT	190	67.8 (50.3–NR)	NR (48.8–NR)	18.3 (12.0–24.2)	33.1 (24.3–40.9)
CAR T	420	18.3 (13.5–22.9)	26.4 (22.6–31.9)	42.2 (36.4–47.5)	59.7 (52.0–66.2)
Post-CAR T^a^	93	4.8 (4.3–6.9)	23.3 (15.2–28.0)	83.9 (74.4–89.9)	^b^

*1L* first line, *2L* second line, *3L* third line, *CAR T* chimeric antigen receptor T-cell, *CI* confidence interval, *LOT* line of therapy, *NR* not reached, *R-CHOP* rituximab, cyclophosphamide, doxorubicin, and vincristine (with or without corticosteroids), *SCT* stem cell transplant, *TTNT* time to next treatment or death, *OS* overall survival.

^a^Defined as the subsequent LOT following CAR T infusion, among patients with continuous enrollment from the infusion date through at least 29 days after the start of next LOT.

^b^The patient count (≤10) was too small to reliably calculate the rate.

#### OS and TTNT by treatment

**Fig. 5:**
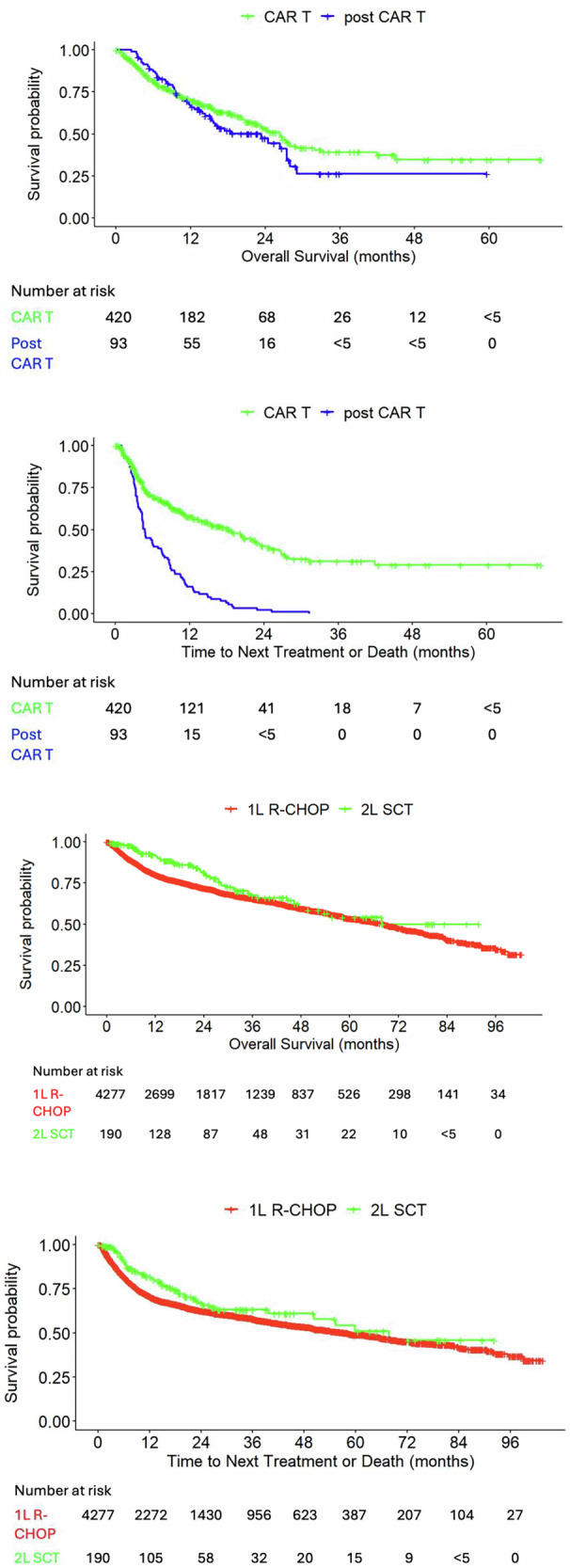
OS and TTNT by treatment. Per Optum’s deidentification policies, patient counts <5 were masked. 1L first line, 2L second line, CAR T chimeric antigen receptor T-cell, OS overall survival, R-CHOP rituximab cyclophosphamide doxorubicin and vincristine (with or without corticosteroids), SCT stem cell transplant, TTNT time to next treatment or death.

The median OS was 68.2 months (95% CI, 63.3–72.7) for 1L R-CHOP, not reached (NR) for 2L SCT, 26.4 months (95% CI, 22.6–31.9) for CAR T, and 23.3 months (95% CI, 15.2–28.0) post-CAR T (Table 3 and Fig. 5). The median TTNT was 58.0 months (95% CI, 50.9–65.6) for 1L R-CHOP, 67.8 months (95% CI, 50.3–NR) for 2L SCT, 18.3 months (95% CI, 13.5–22.9) for CAR T, and 4.8 months (95% CI, 4.3–6.9) post-CAR T (Table 3 and Fig. 5).

#### Treatment failure rates

The overall 1L treatment failure rate was 36.0% (95% CI, 34.9%–37.2%) at 12 months of follow-up, increasing to 44.8% (95% CI, 43.5%–46.1%) by 24 months (Table 3). At 12 months, the failure rate was 29.2% (95% CI, 27.8%–30.7%) following 1L R-CHOP, 18.3% (95% CI, 12.0%–24.2%) following 2L SCT, and 42.2% (95% CI, 36.4%–47.5%) following CAR T (any LOT). By 24 months, these rates increased to 37.5% (95% CI, 35.8%–39.1%) for 1L R-CHOP and 33.1% (95% CI, 24.3%–40.9%) for 2L SCT. The treatment failure rate for the subsequent LOT after CAR T was 83.9% (95% CI, 74.4%–89.9%) at 12 months, rising to 97.8% (95% CI, 91.5%–99.5%) at 24 months. Survival rates by LOT and treatment at 1-, 3-, and 5-year follow-up are summarized in Supplementary Table 3.

## Discussion

This large retrospective cohort analysis describes real-world patient characteristics and highlights evolving treatment patterns and persistent challenges in managing DLBCL. While R-CHOP-based regimens remained the predominant choice for 1L therapy during the study period, treatment options in 2L and 3L evolved substantially over time. By the end of June 2024, over one-third of 2L and 3 L therapies included novel options, such as pola, tafa, CAR T, and other targeted agents. This study revealed that despite the increasing adoption of these novel therapies, substantial unmet needs persisted, as treatment failure rates remained high within 12 months of 1L R-CHOP (29.2%) and 2L SCT (18.3%). The challenges were most pronounced among patients with relapsed or refractory DLBCL requiring later LOT, especially when current CAR T therapies failed to provide effective disease control. During the study period, both OS and duration of treatment response, as measured by TTNT, declined rapidly for patients receiving 2L and 3L therapies.

These findings underscore the urgent need for more effective and durable frontline treatments to improve long-term outcomes and reduce disease progression, and are consistent with previous research [11, 12, 15], including a recent retrospective cohort analysis of treatment patterns and outcomes of patients with DLBCL by Sineshaw et al. using the COTA database, and another real-world analysis of Medicare patients by Garg et al. [11, 12]. Both studies by Sineshaw et al. and Garg et al. identified the dominance of R-CHOP as 1L therapy (Sineshaw et al. study, 63.6%; Garg et al study, 61.2%) for DLBCL, the lack of established standard regimens in later LOT, and the worsening survival outcomes with later LOT.

However, the median real-world TTNT and real-world OS for 1L, 2L, and 3L in this study notably differ from those reported by Sineshaw et al. and Garg et al. [11, 12]. In this study, the median TTNT for 1L, 2L, and 3L was 36.1 months (95% CI, 32.5–38.9), 10.6 months (95% CI, 9.6–12.5), and 7.8 months (95% CI, 6.9–9.3), respectively; the OS for 1L, 2L and 3L was 58.1 months (95% CI, 54.2–60.9), 30.0 months (95% CI, 27.0–33.0), and 18.2 months (95% CI, 15.2–20.9), respectively. In comparison, the study by Sineshaw et al. reported a median TTNT of NR for 1L, 6.3 months (95% CI, 5.0–8.5) for 2L, and 4.3 months (95% CI, 3.6–6.3) for 3L; and a median OS of NR for 1L, 29.2 months (95% CI, 17.3–NR) for 2L, and 11.0 months (95% CI, 7.4–20.8) for 3L [11]. In Garg et al’s study, the authors did not assess TTNT but reported a median OS of NR for 1L, 19.9 months (95% CI, 17.6–23.2) for 2L, and 9.8 months (95% CI, 8.3–12.3) for 3L [12].

These differences may stem from variations in data sources, patient populations, and LOT algorithms used. While Garg et al’s study included 11,880 Medicare beneficiaries and used the Chronic Conditions Warehouse fee-for-service Medicare Part A, B, and D claims data from 2014 to 2019, Sineshaw et al.’s study used COTA data and identified 1347 patients from January 1, 2016, to March 31, 2021 (cutoff date for follow-up, December 8, 2021), this study included 9875 patients with both commercial and Medicare Advantage health plans and used more recent data (data cutoff date, June 30, 2024). Consequently, the findings from this study are likely to be more nationally representative, reflecting current treatment patterns, and more generalizable to broader patient populations in the US.

This study also revealed high treatment failure rates at 12 and 24 months after treatment initiation, with failure rates increasing substantially across later LOT. This finding aligns with the study by Sineshaw et al., which reported 23% of patients experienced disease progression or relapse within 12 months of 1L initiation. Additionally, this study observed a high failure rate for CAR T therapy, with a failure rate of 42.2% at 12 months and 59.7% at 24 months. For patients who went on to receive a subsequent LOT following CAR T, failure rates sharply increased to 83.9% at 12 months and 97.8% at 24 months, underscoring the lack of effective treatment options beyond CAR T.

To our knowledge, this study is the largest real-world analysis of patients with DLBCL treated with CAR T conducted to date. Our findings show a steady increase in CAR T use in 3L setting, with utilization rising from 8.3% in 2018 to 19.5% by June 30, 2024, following the FDA’s October 2017 approval of axicabtagene ciloleucel [16], the first CAR T therapy for DLBCL. In comparison, uptake in 2L setting has been slower, increasing from 0.7% to 10.1% over the same period. This slower adoption may reflect the later approval of CAR T for 2L use and associated logistical challenges. Axicabtagene ciloleucel was approved for 2L treatment in April 2022 for adults with large B-cell lymphoma that is refractory to 1L chemoimmunotherapy or relapses within 12 months of completing 1L chemoimmunotherapy treatment [17].

Findings from this study also highlight the lack of a standard approach to post-CAR T treatment. This observation is consistent with the study by Jalbert et al, which reported a wide range of therapies used in patients requiring additional treatment after CAR T, including immunotherapy, radiation therapy, chemotherapy, targeted therapy, and SCT [13].

This study is subject to limitations inherent to administrative claims data, including potential coding errors and incomplete information, as claims data are primarily intended for billing purposes. Incomplete capture of specific medications may be particularly prevalent in inpatient settings, where antineoplastic treatment may be recorded using generic drug administration codes or bundled payments without identifying specific drugs administered. The prescription claim date represents the date a medication is filled, which may not align with the actual treatment start date; however, it is assumed to approximate the initiation of treatment. Additionally, the data used in this study were derived primarily from a single large US insurance company, which may limit the generalizability of these findings to the broader US patient population. Furthermore, LOT and outcomes, such as TTNT, were determined using a study-specific LOT algorithm. This algorithm may differ from those used in other studies and might not fully reflect patients’ actual treatment regimens, potentially affecting LOT classification and associated outcomes. The 6-month washout period used to identify newly diagnosed DLBCL patients may have been insufficient to exclude prevalent cases, potentially impacting LOT derivation. Furthermore, death dates in the database may be incomplete or misaligned with actual death dates, which could potentially affect the accuracy of OS assessment. Finally, the relatively short follow-up period and loss of follow-up due to factors such as insurance switching may have limited the ability to fully capture long-term outcomes, potentially affecting the study findings and conclusions.

Despite these limitations, this study illustrates the evolving treatment patterns for DLBCL in recent years, highlights the high treatment failure rates associated with available therapies, and underscores the substantial unmet medical needs of patients with this disease.

## Conclusions

Despite the rapid uptake of novel agents in 2L and 3L, the findings from this study indicate substantial unmet medical needs for patients with DLBCL. The persistently poor outcomes in later LOT underscore the urgent need for more effective and durable frontline and relapsed or refractory treatment options to reduce further disease progression.

## Supplementary information

Supplementary material

## Data Availability

These data were made available by Optum and used under license for the current study and are not publicly available. The data are accessible to other researchers by contacting Optum [18].
