# Supplementary material for: Real-world treatment patterns and clinical outcomes among patients with diffuse large B-cell lymphoma in a US healthcare claims database
Source: Blood Cancer J. 2025 Dec 5;16(1):14. doi: 10.1038/s41408-025-01412-8 (PMC12804711; doi:10.1038/s41408-025-01412-8)
Supplement: Supplementary file 1 — Supplementary material [file 41408_2025_1412_MOESM1_ESM.pdf]

Supplementary material

Supplementary Table 1. Demographic and clinical characteristics of patients with DLBCL treated with CAR T

	**CAR T (any LOT)[a]** **n = 420**
**Demographics**	
**Age at index, years**	
Mean (SD)	67.7 (11.4)
Age category, n (%)	
<65 years	119 (28.3)
65-69 years	74 (17.6)
70-74 years	106 (25.2)
≥75 years	121 (28.8)
**Sex, n (%)**	
Male	243 (57.9)
Female	177 (42.1)
**Race, n (%)**	
White	280 (66.7)
Black	23 (5.5)
Asian	14 (3.3)
Other/unknown	103 (24.5)
**US region, n (%)**	
South	147 (35.0)
Northeast	78 (18.6)
Midwest	116 (27.6)
West	77 (18.3)
Unknown	2 (<1)
**Payer type, n (%)**	
Commercial	124 (29.5)
Medicare advantage	296 (70.5)
**Clinical characteristics**	

**QCCI**	
Mean (SD)	5.2 (2.7)
Median	5.0
Comorbidities, n (%)	
Infection	342 (81.4)
Anemia	320 (76.2)
Hypertension	266 (63.3)
Fatigue or asthenia	248 (59.0)
Neutropenia	207 (49.3)
Thrombocytopenia	172 (41.0)
Anxiety or depression	158 (37.6)
Coronary artery disease	141 (33.6)
Obesity	116 (27.6)
Leukopenia	56 (13.3)
**Time from first observed diagnosis to index date, months**	
Mean (SD)	19.8 (15.9)
Median	14.5
**Follow-up time, months**	
Mean (SD)	13.1 (12.2)
Median	9.4

Abbreviations: CAR T, chimeric antigen receptor T-cell; DLBCL, diffuse large B-cell lymphoma; LOT, line of therapy; QCCI, Quan-Charlson Comorbidity Index; SD, standard deviation.

[a]May include patients with multiple CAR T LOT.

Supplementary Table 2. 1L, 2L, and 3L treatment regimens from January 1, 2016 to June 30, 2024

Regimen/Year	2016	2017	2018	2019	2020	2021	2022	2023	2024[a]
**1L**									
R-CHOP	65.3%	62.1%	61.7%	65.2%	62.3%	67.3%	67.3%	62.1%	49.4%
CIT (non R-CHOP)	13.4%	17.3%	15.4%	14.0%	14.2%	11.2%	10.9%	8.8%	11.9%
R monotherapy	11.2%	11.0%	11.7%	9.9%	12.4%	10.2%	9.5%	9.4%	11.2%
Chemotherapy	7.0%	5.7%	7.7%	6.9%	6.5%	5.9%	6.0%	3.6%	4.9%
Pola + R-CHP	0%	0%	0%	0%	0%	0%	1.4%	10.5%	16.4%
Other	1.3%	1.8%	1.1%	1.2%	1.1%	2.3%	1.3%	0.8%	1.1%
SCT	1.7%	1.2%	1.8%	0.8%	0.9%	0.3%	0.4%	0.4%	1.4%
Pola +/- other	0%	0%	0%	0%	0.4%	0.4%	0.6%	2.1%	1.1%
Immunotherapy	0.2%	0.7%	0.0%	1.4%	0.8%	0.5%	1.2%	1.0%	1.3%
R-squared	0.0%	0.1%	0.5%	0.5%	0.9%	0.8%	0.2%	0.4%	0.2%
CAR-T	0.0%	0.0%	0.1%	0.1%	0.3%	0.6%	0.6%	0.7%	1.3%
Tafa +/- other	0.0%	0.0%	0.0%	0.0%	0.1%	0.4%	0.6%	0.2%	0.0%
**2L**									
R-CHOP	31.6%	19.6%	18.2%	16.0%	18.3%	19.5%	15.0%	11.0%	10.6%
CIT (non R-CHOP)	36.0%	36.7%	36.1%	33.8%	30.1%	22.5%	23.6%	15.6%	14.0%
R monotherapy	2.6%	5.5%	9.3%	10.6%	12.2%	6.0%	6.7%	7.1%	7.2%
Chemotherapy	11.4%	14.1%	20.1%	16.0%	13.6%	15.9%	11.0%	14.3%	10.1%
Pola + R-CHP	0.0%	0.0%	0.0%	0.0%	0.0%	0.0%	0.3%	3.7%	6.3%
Other	4.4%	4.0%	6.3%	5.5%	6.4%	6.0%	4.0%	3.9%	6.8%
SCT	11.4%	13.6%	5.6%	8.9%	6.4%	6.3%	8.0%	5.7%	4.3%
Pola +/- other	0.0%	0.0%	0.0%	1.7%	5.2%	8.2%	15.3%	14.9%	10.6%
Immunotherapy	0.9%	4.0%	2.2%	2.7%	2.0%	3.6%	4.6%	6.2%	8.7%
R-squared	1.8%	2.5%	1.5%	2.7%	3.8%	3.0%	1.3%	0.7%	3.9%
CAR T	0.0%	0.0%	0.7%	2.0%	1.7%	1.6%	4.3%	11.7%	10.1%
Tafa +/- other	0.0%	0.0%	0.0%	0.0%	0.3%	7.4%	5.9%	5.1%	7.2%
**3L**									
R-CHOP	0.0%	2.6%	1.2%	2.2%	2.4%	1.5%	2.0%	1.3%	0.0%
CIT (non R-CHOP)	23.8%	33.3%	32.1%	25.6%	15.9%	11.8%	10.5%	7.8%	11.7%
R monotherapy	4.8%	2.6%	4.8%	11.1%	8.7%	8.1%	7.2%	9.1%	6.5%
Chemotherapy	19.0%	23.1%	15.5%	11.1%	16.7%	11.0%	5.9%	15.6%	3.9%
Pola + R-CHP	0.0%	0.0%	0.0%	0.0%	0.0%	0.0%	0.0%	0.0%	2.6%
Other	4.8%	15.4%	14.3%	8.9%	7.1%	11.8%	7.2%	5.8%	9.1%
SCT	33.3%	15.4%	15.5%	15.6%	11.9%	10.3%	11.1%	3.2%	5.2%
Pola +/- other	0.0%	0.0%	0.0%	3.3%	15.1%	13.2%	13.7%	11.7%	5.2%
Immunotherapy	4.8%	7.7%	3.6%	2.2%	3.2%	6.6%	7.2%	13.6%	22.1%
R-squared	9.5%	0.0%	4.8%	4.4%	5.6%	2.2%	0.7%	1.9%	7.8%
CAR T	0.0%	0.0%	8.3%	15.6%	11.9%	16.2%	24.2%	23.4%	19.5%
Tafa +/- other	0.0%	0.0%	0.0%	0.0%	1.6%	7.4%	10.5%	6.5%	6.5%

Abbreviations: 1L, first line; 2L, second line; 3L, third line; CAR T, chimeric antigen receptor T-cell; CIT, chemoimmunotherapy; DLBCL, diffuse large B-cell lymphoma; LOT, line of therapy;

Pola, polatuzumab vedotin; R-CHOP, rituximab, cyclophosphamide, doxorubicin, and vincristine, with or without corticosteroids; R-CHP, rituximab, cyclophosphamide, and doxorubicin (with or without corticosteroids); R-squared, rituximab and lenalidomide; SCT, stem cell transplant; Tafa, tafasitamab.

[a]January 1 to June 30, 2024.

Supplementary Table 3. Survival rate by LOT and treatment at 1-, 3-, and 5-year follow-up

	**Survival rate, %, median (95% CI)**		
	**1-year**	**3-year**	**5-year**
**LOT**			
1L	77.3 (76.3-78.3)	60.8 (59.5-62.2)	48.5 (46.9-50.2)
2L	67.4 (65.5-69.4)	46.1 (43.7-48.5)	36.5 (33.8-39.3)
3L	58.9 (55.5-62.5)	35.2 (31.4-39.5)	23.3 (18.6-29.1)
**Treatment**			
1L R-CHOP	80.2 (78.9-81.4)	65.5 (63.9-67.3)	53.4 (51.3-55.7)
2L SCT	91.4 (87.1-95.8)	67.4 (59.1-77.0)	54.2 (43.9-66.9)
CAR T (any LOT)	70.0 (65.3-75.1)	39.1 (32.0-47.9)	-*
Post CAR T (any LOT)	66.0 (56.7-76.7)	-*	-*

Abbreviations: 1L, first line; 2L, second line; 3L, third line; CAR T, chimeric antigen receptor T-cell; CI, confidence interval; LOT, line of therapy; R-CHOP, rituximab, cyclophosphamide, doxorubicin, and vincristine (with or without corticosteroids); SCT, stem cell transplant.

*The patient count (≤10) was too small to reliably calculate the rate.
